# Seasonal Dynamics in Bacteriological and Physicochemical Water Quality of the Southern Gulf of Lake Tana

**DOI:** 10.1155/2022/7317702

**Published:** 2022-09-27

**Authors:** Baye Sitotaw, Belayneh Daniel, Mulugeta Kibret, Workiye Worie

**Affiliations:** Bahir Dar University, Department of Biology, P.O.Box 79, Bahir Dar, Ethiopia

## Abstract

Freshwater lakes are important sources of water for domestic, agricultural, and industrial uses. Lake Tana offers a range of ecosystem services to the surrounding communities. However, this lake is facing deterioration in water quality due to pollution caused by anthropogenic influences. Therefore, regular monitoring of key water quality parameters is critical to understanding the water quality status of the lake. This study aimed to assess the seasonal fluctuation of water quality of the Southern Gulf of Lake Tana using indicator bacteria and some physicochemical parameters. A total of 48 water samples were collected in dry and wet seasons from eight sites in the study area. Total coliforms (TC), faecal coliforms (FC), and some physicochemical parameters (pH, temperature, turbidity, electrical conductivity, total dissolved solids, dissolved oxygen, biochemical oxygen demand, ammonium, nitrate, and phosphate) were determined following standard methods. The results revealed that coliform counts were above the permissible level set by Ethiopian Standards and varied significantly among sites (*P* < 0.05). FC counts ranged from 1 to 1600 MPN/100 ml (with the lowest and highest mean value of 1 at site 8 to 1076.5 ± 3.1 at site 4) and again TC counts ranged from 1 to 1600 MPN/100 ml (with a mean value of 4.8 ± 1.81 at site 6 to 1600 at site 4 and site 8). The findings also confirmed that the highest counts of coliforms were observed during the wet season. The high counts are attributed to the discharge of human excreta and animal wastes during the rainy season from the different anthropogenic activities near the Gulf. Significant variations in most of the physicochemical parameters were also observed between sites and seasons. FC and biochemical oxygen demand (BOD_5)_ in most sites did not meet the EPA standard for surface water. Corrective measures are highly recommended for anthropogenic activities driving high pollution loads in the lake.

## 1. Introduction

Freshwater is a vital resource for human survival and development. It serves multiple purposes including drinking, transportation, irrigation, and recreation, as well as provides habitat for a tremendous number of species. However, it is a very scarce resource, constituting less than 3% of all kinds of waters on the Earth, where more than 97% is saline and not suitable for most life forms [1]. Surprisingly, the majority (69%) of freshwater is unavailable (locked up in glaciers, polar ice caps, atmosphere, and soil; highly polluted; or lies too far under the Earth's surface to be extracted at an affordable cost). The most accessible freshwaters comprise lakes, rivers, and streams, and they account for less than 0.01% of the total volume of water [1].

Freshwater lakes (FWLs) contain about 87% of all liquid fresh surface water and provide the most easily accessible source of freshwater for humans and habitat for much of the planet's aquatic biological diversity [1]. FWLs, thus, constitute the major surface water resources for domestic, agricultural, and industrial purposes and are essential for the development of human civilization. They are also considered ecological barometers of the health of a region because lakes are playing an important role in regulating the microclimate of any region, thereby influencing the life of the people adjacent to them.

The integrity of FWLs depends on a complex interaction among physical, chemical, and biological components as indicated in Bhateria and Jain [2]. The utility of surface water, such as in lakes, for various purposes, is governed by the physicochemical and biological quality of the water. The quality of freshwater lake water is a critical factor affecting human health and welfare of the nearby communities [3]. Unfortunately, FWLs are highly vulnerable to disturbance and pollution owing to their accessibility and suitability [2, 4, 5]. Several cities, industrial infrastructures, and agriculture complexes have been built up near lakes and other water bodies. As a result, unwise use of water resources is common and has deteriorated lake and river water quality [2].

Surface water quality encompasses a wide range of conditions that are part of the aquatic environment. Excessive nutrient and toxic substances carrying runoff from point and nonpoint sources, where both of them are human-related activities, increase the pollutants of various forms and types of the water and cause intense surface water quality degradation [2]. Several chemical and physical parameters influence the quality of lake water and hence its ecosystem [2, 6]. The dynamics of these parameters depend on both natural phenomena (the climatic and geological conditions in the basin and the nature of the lake itself) and multitudes of anthropogenic activities [2]. Thus, the health of any lake system depends upon the nature of that lake and its exposure to various environmental factors. Consequently, any change in physical, chemical, and biological variables can affect the aquatic biota and humans in a variety of ways [7]. It is thus essential to assess the quality of surface water, such as lakes, at certain time intervals whenever possible.

Several studies have shown multiple burdens to freshwater lakes from nearby cities, industries, and agricultural activities [2]. For instance, studies by Karakoc et al. [8] in Eymir and Mogan lakes (Turkey), Najar et al. [9] in three lakes of Kashmir (India), Islam et al. [10] in some urban lakes of the rapidly urbanizing city of Dhaka (Bangladesh), Yang et al. [11] in six urban lakes of central China, and Henny et al. [12] in urban lakes of megacity Jakarta have reported severe pollution loads from the surrounding urban areas. Particularly, abundant antibiotic-resistant bacteria in shallow urban lakes after stormwater events [13], intestinal pathogens, and microbial pollution associated with urban pressure and different land use types have been reported elsewhere [14–17].

Ethiopia is a tropical country, which possesses many forms of aquatic ecosystems including several lakes that are of great scientific interest and economic importance. Lake Tana, with the largest surface area, is a freshwater body in Ethiopia. This lake has crucial importance for serving multiple purposes (transportation, recreation, bathing, drinking, and irrigation) for the surrounding community. It has also been identified as a growth corridor by the federal government and as a biosphere reserve by UNESCO in 2014 [18].

Although Lake Tana is a large water body, it is a shallow lake and there is a dense human settlement in its catchment. As a result, Lake Tana is highly prone to pollution due to human-induced activities as well as the nature of the lake. It receives urban surface runoff, industrial, and agricultural wastes from the catchment and thus the water quality has deteriorated over time. Agriculture inputs (pesticides, fertilizers, and organic manure) and wastewaters from different institutions, industries, residents, recreation centers, and street runoff are directly loaded into the lake [19]. Particularly, there is a high population density and intense anthropogenic activity in the Southern Gulf of Lake Tana compared to other parts, and the water quality deterioration can be higher here than in any other part of the lake. Few studies reported the pollution load in this section of the lake. For instance, Wondie [20] reported the impact of urban stormwater runoff and domestic waste effluent on the water quality of Lake Tana, and Goshu et al. [21] demonstrated a pollution level higher in sites close to the Bahir Dar city than farther sites. Therefore, assessing the quality of lake water using indicator microorganisms and physicochemical parameters in different seasons is of pivotal importance in generating scientific information on the current pollution status of the lake. Regular follow-up of water quality in lakes is vital for assuring safe water for drinking, bathing, fishing, and various agricultural activities. With water quality analysis at some time intervals, long-term trends and short-term changes, which are indicators of environmental health and changes in the water catchment area, can be monitored. Directives such as the EU's Water Framework Directive or the USEPA Clean Water Act request regular information about the ecological status of all lakes larger than 50 ha.

Few studies reported the impact of anthropogenic faecal pollution of Lake Tana [19, 22]. However, previous studies did not include seasonal data to understand the dynamics of water quality in the Southern Gulf of Lake, and previous studies covered a few sites. Above all, regular monitoring of water quality in such highly vulnerable systems is vital to understand the current water quality status and hence enforce the appropriate measures to reduce the pollution load. This study aimed to assess the seasonal fluctuation of water quality in the Southern Gulf of Lake Tana using pollution indicator microorganisms and some selected physicochemical parameters.

pH, temperature, dissolved oxygen, conductivity, and turbidity are basic parameters to life within aquatic systems. Changes in these parameters can be observed as impacts on the flora and or fauna in a given water body. Other constituents of concern such as coliforms (total and faecal coliforms*, E.coli*), nitrate, nitrite, ammonia, phosphate, sulfate, total dissolved solids, total suspended solids, and 5 days biological oxygen demand (BOD_5_) can be included in the water quality analyses program. In this study, we measured twelve of the sixteen water quality parameters listed above. Moreover, water quality index was calculated which can be used to reflect the overall and ongoing condition of the water. As with most monitoring programs, an index will not usually show the effect of spills, and other such random and transient events, unless these are relatively frequent or long-lasting. In this study, we calculated the water quality index for each site to understand the overall pollution status of the lake and complement the other water quality criteria considered.

## 2. Materials and Methods

### 2.1. Description of the Study Area

**Figure 1:**
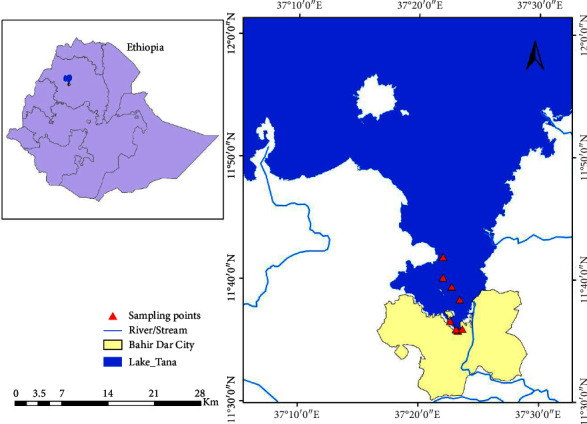
Map showing sampling sites at the Southern Gulf of Lake Tana.

The study was conducted in the Southern Gulf of Lake Tana, which is close to Bahir Dar city, 480 km northwest of Addis Ababa (the capital of Ethiopia) (Figure 1). It is located between 11°35′ to 12°17′ N latitude and 37°37′ *E* longitude and at an altitude of 1800 meters above sea level on a basaltic plateau.

Lake Tana, which is the source of the Blue Nile River, is the largest lake by surface area (3156 km^2^) in Ethiopia, contributing about 50% of the total freshwater resources of the country. It is a shallow lake with a mean depth of 8 m and a maximum depth of 14 m.

The Lake Tana region experienced changes in the environmental balance forced partly by climate change and mostly by the persistence of unsustainable production and consumption systems [23]. The area has warm temperature where the mean annual temperature is 13.5 to 27.7°C; the mean annual rainfall is about 1500 mm, of which 54% fall in July and August when the rainfall can reach 250-300 mm per month. The seasonal rainfall causes the lake to fluctuate regularly. Lake Tana and its adjacent wetlands provide directly and indirectly a livelihood for more than 500,000 people [24], and about three million people live in the catchment. The population density is high in the areas to the northeast and south of Lake Tana, with the highest in the north and south of Lake Tana [23]. Of the total area (15.000 km^2^) of Lake Tana catchment, about 55% is cultivated, 21.06% is water area, 10.38% is grassland, 1.6% is wetland/swampy area, and 0.39% is natural forest [25]. The cultivated area is used for the growing of common crops.

Seven large permanent rivers and about 40 small seasonal rivers feed the lake with sediment, soil, and pollutants from the catchment. Specific to the Southern Gulf Lake Tana, the urban pressure from Bahr Dar city is high, where several hotels, institutes, recreational centers, and small-scale agricultural activities release multitudes of wastes directly to the lakeshore.

### 2.2. Study Design and Periods

The study was seasonal, aiming to evaluate the water quality dynamics of the Southern Gulf of Lake Tana in two major seasons (dry and wet) in the period from February to August 2018. Sampling in May was omitted as there were sporadic rains that cannot be categorized as the wet or dry season.

### 2.3. Selection Criteria of Sampling Sites

A total of eight sampling sites were purposely selected based on the expected human impact. Four of the sampling sites were from more influenced areas by high human activity and exposure to pollution. These four sites were identified based on proximity and access, anthropogenic activity (lakeshore recreation practices, open bathing, and laundering activities), stormwater loading, human settlement, and the extent of littering waste into the lake. The remaining four were from areas relatively less impacted by human activities that can represent reference sites (Table 1). The number of sites is higher than the recommended number by United Nations Environmental Program (UNEP) Water Quality Monitoring [26] for lakes with a given surface area.

### 2.4. Sample Collection

The sample collection procedures were based on APHA [27]. The samples for microbial and physicochemical parameters were collected on the same day from the surface of the lake to achieve consistency in sampling. Accordingly, a total of 48 samples were collected from the surface (about 30 cm in depth). Temperature, dissolved oxygen, pH, TDS, and conductivity were measured on-site at the time of sampling. Water samples for the microbial quality analyses were collected using sterilized 200 ml glass bottles, but 500 ml acid-washed polyethylene bottles were used to collect water samples for the analyses of the remaining chemical parameters. Before the collection of the samples, the containers were thoroughly rinsed with lake water. The samples were transported to the laboratory using icebox, and analyses were started within 2 hours of collection. The sampling date was monthly based and the time was between 7 and 8 am.

### 2.5. Determination of Coliforms

The coliform count was calculated using the multiple tubes most probable number (MPN) method. In doing so, fifteen culture tubes were used per sample where five tubes containing sterile 10 ml double strength and ten tubes containing 10 ml single strength MacConkey broth (HiMedia, India), and all tubes were fitted with inverted Durham tubes. Then, 10 ml of the water sample was aseptically dispensed into each of the first five culture tubes containing the double strength MacConkey broth. One milliliter of the sample was inoculated into each of the five culture tubes containing sterile single strength MacConkey broth, while a 0.1 ml sample was inoculated into the remaining five tubes. The tubes were gently shaken to distribute the sample uniformly throughout the medium and incubated at 37°C for 24 hours. After 24 hours of incubation, the cultures were observed for acid and gas production.

For the confirmatory test, a loop full of culture from test tubes that showed gas production was transferred to the brilliant green lactose bile (BGLB) broth tube and incubated for 24 to 48 hours at 35°C. Tubes showing gas production inside Durham tubes, growth/turbidity/,and color change of purple MacConkey broth to yellow were considered as positive for total coliforms (TC). Finally, the results were reported as the most probable number (MPN) per 100 milliliters of water sample. The above procedure was repeated for Faecal Coliforms (FC), but here, the tubes were incubated at 44.5°C for both presumptive and confirmatory tests.

### 2.6. Determination of Chemical Parameters

Nitrate (NO_3_^−^) and ammonium (NH_4_^+^) were determined following the procedures in APHA [27]. Five days biological oxygen demand (BOD_5_) was determined by using BOD OxiTop meter as described in Yuan et al. [28].

### 2.7. Data Analysis

Statistical analyses were performed using SPSS software version 21 (SPSS Inc, Chicago, USA). Coliform counts and measured values of physicochemical parameters were compared with the WHO or EPA guidelines for surface water quality. The values were also compared among sites and between the two seasons for significant differences using parametric or nonparametric statistical tests. A significance level of *P* ≤ 0.05 was applied for all statistical tests. Moreover, the water quality index (WQI) was calculated for impacted and reference sites, as well as for the dry and wet seasons, following the procedures in CCME [29].

### 2.8. Limitations

Conducting water quality research on Lake Tana requires as many representative sites as possible with sufficient time to reach more sound scientific conclusions. This study was conducted only in eight sites (four sites giving particular emphasis on impacted areas and the rest of the four sites as reference sites), and more physicochemical parameters, detection of common pathogenic microorganisms, and heavy metals were not tested due to time and financial issues.

## 3. Results

### 3.1. Faecal and Total Coliform Counts

In this study, there were significant variations in total coliform (TC) (*χ*^2^ = 92; *P* < 0.05) and faecal coliform (FC) (*χ2* = 79, *P* < 0.05) counts among all sites. As indicated in Table 2, coliform counts were significantly higher in the impacted sites than in the reference sites during the dry season. However, coliform counts did not differ between impacted and reference sites in the wet season. Moreover, regardless of the status of the sites, wet season samples were found to be more contaminated with coliforms than those of dry season samples (Table 2), that is, there was no statistical difference in coliform counts between impacted and reference sites in the wet season except for TC counts that did not show a statistical difference between dry and wet season in the impacted sites.

Total coliform counts in all sites and both seasons, and faecal coliform counts in most sites and dry season were found to be within the permissible level recommended by EPA (Table 2). However, in the wet season, 55 to 66.7% of water samples from impacted sites and 22 to 58% of water samples from reference sites were found to have faecal coliform counts exceeding the recommended values (Table 3).

### 3.2. Physicochemical Parameters

As indicated in Table 4, five out of ten physicochemical parameters were found to exceed the respective limit values in some sites, and except for phosphate, all of these samples were those collected during the wet season. Particularly, the level of BOD_5,_ which ranged from 2.3 to 23 mg/l, was above the permissible limit (5 mg/L) in 50% of the samples (all in the wet season). The measured pH and ammonium were also higher in the wet season than those in the dry season.

As shown in Table 5, the water quality indices for all sites, except reference sites in the dry season, were categorized as fair or marginal.

## 4. Discussion

Freshwater lakes, which serve multiple purposes for humans and the biodiversity that supports them, have been subjected to a continuing burden mainly due to human activities in the vicinity. Several forms of human activities generate multitudes of pollutants that enter the lake. The problem is intensified in lakes on which the livelihood of local communities depends to large extent [30] when there is unmanaged urbanization in the lake catchment [20] as well as no buffer zones around the lake [31]. Moreover, shallow lakes are more vulnerable to pollution as their diluting capacity is limited. Lake Tana, which supports millions of lives, is a shallow lake exposed to the aforementioned problems. With these views, this study aimed to assess the seasonal fluctuation and spatial distribution of selected pollutants in the Southern Gulf of Lake Tana from water samples collected from impacted (near the residential/and or business area) and reference sites (open water relatively far away from the residential area).

Based on the measured bacteriological water quality parameters, all water samples collected during the wet and dry seasons conform to the guideline values set for surface water quality in terms of total coliform (TC) counts (Table 3). Similarly, faecal coliform (FC) counts in all samples collected in the dry season were within the recommended values for surface water quality. A previous study conducted in the same lake reported TC counts of about 90 to 200 CFU/100 ml, which were by far lower than the recorded values in the present study (median value of 1600 MPN/ml). However, in the wet season, more than half of the samples in the impacted sites and from 22 to 44% of the samples in the reference sites had FC counts exceeding the recommended value (Table 2). The results evidently indicated that faecal pollution was increased during the wet season, likely as a result of stormwater carrying pollutants into the lake from the residential and/or business areas. It is also shown that the impacted sites had higher counts of FC than the reference sites, which implies the pollutants might be diluted, adsorbed, precipitated, or eliminated by some process as they move further into the lake.

The most important points that need attention are the incidences of pollutants and the aggravating factors (stormwater). In this regard, Lake Tana has been still in a pollution problem showing no progress from previous reports. For example, a study by Wondie [20] demonstrated the magnitude of pollutants due to stormwater from Bahir Dar city area; Goshu et al. [22] found a high level of faecal and physicochemical pollution in a part of Lake Tana near Bahir Dar city compared to farther sites. Thus, the results of this study and previous works have indicated that Lake Tana is under continuous pressure due to pollution from different human activities in the catchment.

Regarding the physicochemical parameters, most of the measured values for the 10 parameters (Table 4) were found to be within the permissible limits, which implies that the pollutants are probably being diluted by the lake water. Indeed, this does not mean the lake is safe as its diluting capacity may deteriorate in the near future unless appropriate measures are taken to reduce the pollution load. However, the biological oxygen demand (BOD_5_), which is a very good indicator of organic pollution, has significantly increased during the wet season in all sampled sites. This further strengthens the above results (coliform loads), on the impact of runoff or stormwater from Bahir Dar city on the water quality of Lake Tana. As high as 20 to 23 mg/L of BOD_5_ was recorded in both impacted and reference sites, showing the strong influence of the incoming runoff likely from the city.

Based on the water quality index determined using the combined parameters, most sites are categorized as *fair* or *marginal,* still showing this section of Lake Tana worth attention in terms of water quality deterioration.

## 5. Conclusion

The results of this study demonstrated that Lake Tana is currently at a high risk of faecal and organic pollution. These pollutants were found to be significantly higher during the wet season, likely due to the stormwater/runoff coming from Bahir Dar city. Delimiting the buffer zone, advancing the waste management system in Bahir Dar city, improving the livelihood of the communities who depend on Lake Tana water, and the practices of wise use of the lake water are highly recommended. In addition, the local communities who rely on this lake for drinking, bathing, and any other purposes should be communicated about the health risks related to the water quality, and finally, the city administration should find out alternatives for communities who used the lake water for a direct purpose.

## Figures and Tables

**Table 1:** Sampling sites and description at the Southern Gulf of Lake Tana, 2021.

Site code	Site description
S1	Near Taitu recreational center and St. George Church
S2	Close to Kuriftu and Grand Hotel
S3	Between Tana Hotel and Shum Abo recreational center
S4	Close to St. Michael Monastery and Referral Hospital
S5 to S8	From pelagic areas of the southern part of Lake Tana where pollution is expected to be minimal

**Table 2:** Median count (standard error) of coliform counts in water samples collected from Southern Gulf of Lake Tana (*n* = 36), 2021.

Coliform	Site	Season	Median (SE^*∗*^)	Range	Mean rank	Mann–Whitney *U* test	*P*-Value (asymp. sign)
FC	Impacted	Dry	14 (9.2)	1-280	53.86	59	≤0.01
	Reference	Dry	1 (0.23)	1-7	20.14		
	Impacted	**Wet**	**1600 (123.7)**	**2-1600**	**40.5**	**505**	**0.86**
	Reference	**Wet**	**350 (120.8)**	**2-1600**	**32.5**		
	Impacted	Dry	14 (9.2)	1-280	23.03	235	≤0.01
	Impacted	**Wet**	1600 (123.7)	2-1600	47.97		
	Reference	Dry	1 (0.23)	1-7	19.575	38.5	≤0.01
	Reference	**Wet**	350 (120.8)	2-1600	53.43		

TC	Impacted	Dry	1600 (70.4)	500-1650	52.68	65	≤0.01
	Reference	Dry	14 (67.06)	1-1650	20.32		
	Impacted	**Wet**	**1600 (46.6)**	**500-1600**	**36.85**	**635.5**	**0.815**
	Reference	**Wet**	**1600 (90.2)**	**2-1600**	**36.15**		
	Impacted	Dry	**1600 (70.4)**	**500-1650**	**30.4**	**543.5**	**0.186**
	Impacted	**Wet**	**1600 (46.6)**	**500-1600**	**33.60**		
	Reference	Dry	14 (67.06)	1-1650	23.57	182.5	≤0.01
	Reference	**Wet**	1600 (90.2)	2-1600	49.43		

*Note.* Impacted sites (sites 1 to 4); Reference sites (sites 5 to 8).

**Table 3:** Compliance of all water samples collected from all sites in the Southern Gulf of Lake Tana with the guidelines in terms of coliform counts, 2021.

Description of samples and coliforms counted	Number of samples	% Of samples exceeding the limit value
All dry season samples tested for TC	72	0
All wet season samples tested for TC	72	0
All dry season samples tested for FC	72	0
Wet season and site 1 tested for FC	9	55.6
Wet season and site 2 tested for FC	9	55.6
Wet season and site 3 tested for FC	9	55.6
Wet season and site 4 tested for FC	9	66.7
Wet season and site 5 tested for FC	9	22.2
Wet season and site 6 tested for FC	9	44.4
Wet season and site 7 tested for FC	9	33.3
Wet season and site 8 tested for FC	9	44.4
Wet season and impacted sites tested for FC	36	58.3
Wet season and reference sites tested for FC	36	36.1

*Note.* EPA 2001 guideline for surface water of FC = 1000, TC = 5000.

**Table 4:** Mean and standard deviation of physicochemical parameters measured at the Southern Gulf of Lake Tana (*N* = 3), 2021.

Site	Par. Sea.	pH	Temp (°C)	Turb(NTU)	E.C(*µ*S/cm)	TDS(ppm)	NH_4_ (mg/l)	NO_3_ (mg/l)	PO_4_ (mg/l)	DO (mg/l)	BOD5(mg/l)
S1	Dry	7.8 ± 01	25 ± 2	27.7 ± 3	203 ± 10.7	88 ± 13.1	0.2 ± 0.1	39.7 ± 5.2	0.06 ± 0.01	6.1 ± 0.5	4.5 ± 0.5
	Wet	7.9 ± 0.05	22.7 ± 1	11 ± 0.4	285 ± 1.5	148 ± 4	0.5 ± 0.2	36.3 ± 3.1	0.38 ± 0.05	4.7 ± 0.2	**11.7** **±** **3.1**

S2	Dry	7.9 ± 0.32	22.8 ± 0	23 ± 0.8	141 ± 11	71 ± 8.4	0.15 ± 0.2	33.2 ± 3.6	1.4 ± 1.2	6.4 ± 0.4	4 ± 1.3
	Wet	8.7 ± 0.1	25.3 ± 1.1	23.1 ± 2	167 ± 0.8	84 ± 0.1	0.16 ± 0.1	13.9 ± 1.7	0.14 ± 0.1	6.7 ± 0.15	**13**

S3	Dry	8.2 ± 0.6	24.9 ± 0.7	22.9 ± 6	174 ± 22	81 ± 6.1	0.1 ± 0.07	26.7 ± 3.7	1.6 ± 0.5	6.2 ± 0.4	4.7 ± 1.5
	Wet	9.3 ± 0.06	24 ± 1.2	19.4 ± 1	146 ± 3.4	73 ± 0.1	0.37 ± 0.02	33 ± 2.5	0.31 ± 0.1	7.7 ± 0.3	**11** **±** **1.7**

S4	Dry	8.4 ± 0.4	25.6 ± 0.5	24.8 ± 4	193 ± 16	86 ± 13.1	0.16 ± 0.07	57.2 ± 8.6	0.58 ± 0.95	6.5 ± 0.5	5
	Wet	8.6 ± 0.2	21.9 ± 1.5	16 ± 0.3	146 ± 0.5	73 ± 0.1	0.59 ± 0.3	27 ± 2.5	0.47 ± 0.1	6.8 ± 0.5	**23** **±** **2.6**

S5	Dry	7.4 ± 0.2	23.5 ± 0.8	16.3 ± 3	123 ± 7.7	61 ± 4.9	0.12 ± 0.03	15 ± 13	0.02 ± 0.02	6.1 ± 0.5	2.8 ± 1.4
	Wet	9.3 ± 0.06	21.3 ± 0.6	18.7 ± 1.5	131 ± 0.1	66 ± 0.4	0.2 ± 0.27	8.1 ± 6	0.04 ± 0.04	7.6 ± 0.5	**5.7** **±** **1.5**

S6	Dry	7.4 ± 0.1	23 ± 0.7	14.9 ± 2	122 ± 9.9	71 ± 14.6	0.08 ± 0.07	4.5 ± 3.9	0.02 ± 0.02	6.7 ± 0.5	2.3 ± 0.6
	Wet	8.99 ± 0.1	22	27 ± 0.5	146.9 ± 3	74 ± 0.3	0.39 ± 0.15	30 ± 1.5	0.32 ± 0.95	7.9 ± 0.5	**17** **±** **2.5**

S7	Dry	7 ± 0.6	25 ± 0.7	18.4 ± 1.2	134 ± 9.6	58 ± 15.6	0.07 ± 0.06	6.1 ± 0.8	0.03 ± 0.03	6.3 ± 0.5	2.3 ± 0.6
	Wet	8.9 ± 0.2	21 ± 1	24.9 ± 0.2	139 ± 1.9	71 ± 0.1	0.32 ± 0.11	17 ± 1.5	0.12 ± 0.05	7.5 ± 0.5	**22** **±** **2**

S8	Dry	7.3 ± 0.9	24 ± 1.3	11.8 ± 1.7	116 ± 6.9	62 ± 6.7	0.03 ± 0.03	4.5 ± 3.9	0.02 ± 0.02	6.9 ± 0.6	4.0±0.5
	Wet	8.9 ± 0.02	20.7 ± 0.6	26.8 ± 0.6	157 ± 47	70.6	0.56 ± 0.01	34 ± 1.5	0.28 ± 0.04	6.7 ± 0.9	**20.5** **±** **3**

*Note.* Par. = parameters; Sea. = season; Temp = temperature; EC = electrical conductivity; Turb = turbidity; TDS = total dissolved solids; DO = dissolved oxygen; BOD = biological oxygen demand; NO_3_ = nitrates; PO_4_ = phosphate; NH_4_ = ammonium; EPA 2001: PO_4_ 0.5; NO_3_ = 50, conductivity = 1000, BOD_5_ = 5, pH = 5.5 to 8.5 for surface water.

**Table 5:** Water quality index (WQI) calculated for the sampled sites at the Southern Gulf of Lake Tana using both bacteriological (coliforms) and physicochemical parameters, 2021.

Major site categories	Site	Season	WQI	Category
Impacted sites	1	Dry		
		Wet	70.7	
		Combined	68.5	Fair
	2	Dry		
		Wet	70.2	
		Combined	68.6	Fair
	3	Dry		
		Wet	70.8	
		Combined	57.6	Marginal
	4	Dry		
		Wet	66.5	
		Combined	56.6	Marginal

Reference sites	5	Dry		
	5	Wet		
	5	Combined	78	Fair
	6	Dry		
	6	Wet		
	6	Combined	67.06	Fair
	7	Dry		
	7	Wet		
	7	Combined	67.5	Fair
	8	Dry		
	8	Wet		
	8	Combined	66.1	Fair

Impacted sites combined	1 to 4	Dry	76.2	Fair
	1 to 4	Wet	53.3	Marginal

Reference sites combined	5 to 8	Dry	95	Excellent
	5 to 8	Wet	69.4	Fair

All sites combined	1 to 8	Dry	78.4	Fair
	1 to 8	Wet	64.4	Marginal

## Data Availability

All data supporting the findings of this study are included in the paper; however, details of the full data may be obtained from the corresponding author on request.

## References

[1] Wetzel G., Robert (2001). *Limnology: Lake and River Ecosystems*.

[2] Bhateria R., Jain D. (2016). Water quality assessment of lake water: a review. *Sustainable Water Resources Management*.

[3] Wang L., Wang Z., Liu J. (2018). Deriving the freshwater quality criteria of BPA, BPF and BPAF for protecting aquatic life. *Ecotoxicology and Environmental Safety*.

[4] Bogardi J. J., Leentvaar J., Sebesvári Z. (2020). Biologia Futura: integrating freshwater ecosystem health in water resources management. *Biologia Futura*.

[5] Dudgeon D. (2019). Multiple threats imperil freshwater biodiversity in the Anthropocene. *Current Biology*.

[6] Papatheodorou G., Demopoulou G., Lambrakis N. (2006). A long-term study of temporal hydrochemical data in a shallow lake using multivariate statistical techniques. *Ecological Modelling*.

[7] Bashir I., Lone F. A., Bhat R. A., Mir S. A., Dar Z. A., Dar S. A. (2020). Concerns and threats of contamination on aquatic ecosystems. *Bioremediation and Biotechnology*.

[8] Karakoç G., Ünlü Erkoç F., Katırcıoğlu H. (2003). Water quality and impacts of pollution sources for Eymir and Mogan Lakes (Turkey). *Environment International*.

[9] Najar I. A., Khan A. B. (2011). Assessment of water quality and identification of pollution sources of three lakes in Kashmir, India, using multivariate analysis. *Environmental Earth Sciences*.

[10] Islam M. N., Kitazawa D., Runfola D. M., Giner N. M. (2012). Urban lakes in a developing nation: drivers, states and impacts of water quality and quantity in Dhaka, Bangladesh. *Lakes & Reservoirs: Science, Policy and Management for Sustainable Use*.

[11] Yang Y., Xu C., Cao X., Lin H., Wang J. (2017). Antibiotic resistance genes in surface water of eutrophic urban lakes are related to heavy metals, antibiotics, lake morphology, and anthropic impact. *Ecotoxicology*.

[12] Henny C., Meutia A. A. (2014). Urban lakes in megacity Jakarta: risk and management plan for future sustainability. *Procedia Environmental Sciences*.

[13] Zhang S., Pang S., Wang P. (2016). Antibiotic concentration and antibiotic-resistant bacteria in two shallow urban lakes after stormwater event. *Environmental Science and Pollution Research*.

[14] Buckerfield S. J., Quilliam R. S., Bussiere L. (2020). Chronic urban hotspots and agricultural drainage drive microbial pollution of karst water resources in rural developing regions. *Science of the Total Environment*.

[15] Yuan T., Vadde K. K., Tonkin J. D. (2019). Urbanization impacts the physicochemical characteristics and abundance of fecal markers and bacterial pathogens in surface water. *International Journal of Environmental Research and Public Health*.

[16] Zhang H., Wang Y., Chen S. (2018). Water bacterial and fungal community compositions associated with urban lakes, Xi’an, China. *International Journal of Environmental Research and Public Health*.

[17] Sharip Z., Fauzi Mohamad M. (2019). Microbial contamination in urban tropical lentic waterbodies and ponds along an urbanization gradient. *Pertanika Journal of Tropical Agricultural Science*.

[18] UNESCO 2014 (2014). *Lake Tana Biosphere Reserve, Ethiopia*.

[19] Ewnetu D. A., Bitew B. D., Chercos D. H. (2013). Determination of surface water quality status and identifying potential pollution sources of lake Tana: particular emphasis on the lake boundary of bahirdar city, amhara region, north west Ethiopia. *Journal of Environment and Earth Science*.

[20] Wondie T. A. (2009). *The Impact of Urban Storm Water Runoff and Domestic Waste Effluent on the Water Quality of Lake Tana and Local Groundwater Near the City of Bahir Dar, Ethiopia*.

[21] Goshu G., Koelmans A. A., de Klein J. J. M. (2011). Water quality of lake Tana basin, upper Blue nile, Ethiopia. A review of available data. *Social and Ecological System Dynamics*.

[22] Goshu G., Byamukama D., Manafi M., Kirschner A. K., Farnleitner A. H. (2010). A pilot study on anthropogenic faecal pollution impact in Bahir Dar Gulf of Lake Tana, Northern Ethiopia. *Ecohydrology and Hydrobiology*.

[23] Birhanu T., Lee R., Zewudie E. Development initiatives and challenges for sustainable resources management and livelihood in the Lake Tana region of Northern Ethiopia.

[24] Singh A. K., Kaur R., Verma S., Singh S. (2022). Antimicrobials and antibiotic resistance genes in water bodies: pollution, risk, and control. *Frontiers in Environmental Science*.

[25] International Fund for Agricultural Develepment (IFAD) (2007). Community-based integrated natural resources management project in Lake Tana watershed-Ethiopia.

[26] Bartram J., Ballance R., World Health Organization & United Nations Environment Programme (1996). *Water Quality Monitoring: A Practical Guide to the Design and Implementation of Freshwater Quality Studies and Monitoring Programs*.

[27] APHA (American Public Health Association) (2005). *Standards Methods for the Examination of Water and Wastewater*.

[28] Yuan S. Y., Chang J. S., Yen J. H., Chang B. V. (2001). Biodegradation of phenanthrene in river sediment. *Chemosphere*.

[29] Canadian Council of Ministers of the Environment (CCME, 2001) (2001). Canadian water quality guidelines for the protection of aquatic life: CCME Water Quality Index 1.0, User’s Manual. *Canadian Environmental Quality Guidelines, 1999*.

[30] Anteneh M. (2017). Demographic characteristics of the lake Tana basin. *Social and Ecological System Dynamics*.

[31] Norris V. O. L. (1993). The use of buffer zones to protect water quality: a review. *Water Resources Management*.

